# PhishVN: A time-stamped Vietnamese URL phishing dataset with impersonation-scenario labels and confidence tiers

**DOI:** 10.1016/j.dib.2026.113195

**Published:** 2026-08-25

**Authors:** Thai Nguyen Vu

**Affiliations:** Campus in Ho Chi Minh City, University of Transport and Communications, Ho Chi Minh City, Vietnam

**Keywords:** Cybersecurity, Social engineering, Brand spoofing, Blacklist, Benchmark, Machine learning, Temporal split, Lexical features

## Abstract

PhishVN is an open phishing-website dataset localised to the Vietnamese context, compiled from public national and community threat feeds and allow-lists. Its core is a *time-stamped, verified* URL table of 18,997 records (2587 phishing from the national feed, 16,410 legitimate), extended by an explicitly-tagged *bronze* expansion stratum, community-reported domains from the ChongLuaDao project (with per-domain first-seen dates reconstructed for ∼50% from public archival traces) and recent OpenPhish URLs, for 53,116 records in total, each described by a 21-feature lexical/infrastructure schema that is *aligned with the CompPhish dataset* to enable cross-dataset study. Phishing URLs are drawn from the National Cyber Security Centre feed (Tin Nhiem Mang); legitimate URLs form three explicitly-tagged strata of increasing difficulty: the certified “trusted organisation” directory (easy, curated), a .vn-filtered Tranco slice (popular sites Vietnamese users actually visit), and a global Tranco sample (hard, traffic-based negatives that break the “non-.vn implies phishing” shortcut), to strengthen external validity. Every record carries an impersonation-*scenario* label (bank, government, tax, e-commerce, telecom, delivery, social, gaming) inferred from brand tokens in the URL, and a *label-confidence tier*(gold/silver/bronze) derived from the source’s verification status. Records carrying a source-attested event date (the national-feed core phishing, the certified organisations and the date-reconstructed half of the bronze stratum) are ordered temporally and split *with grouping by registrable domain*, so that near-duplicate campaign subdomains never span train and test; records whose registrable-domain group carries no attested event date (most of the web-whitelist and top-list negatives, and a small undated remainder of the bronze stratum) are placed into splits by grouped random assignment. We release the datasheet, the full collection and processing pipeline, and reproducible URL baseline detectors reported with multi-seed spreads and bootstrap confidence intervals. A *preliminary* multi-modal companion additionally pairs 868 URLs across both classes (209 phishing, 659 benign) with the rendered page HTML and a screenshot; the text-message and email channels are left to a planned extension.

Specifications Table

**Table:** 

Subject	Computer Science: Security and Privacy
Specific subject area	Phishing-website detection: Vietnamese-targeted URL threat data with impersonation-scenario labels, label-confidence tiers and a temporal split
Type of data	Table (.csv); Text documentation (.md, .txt, .cff). Gated companion: web pages (.html) and images (.pngscreenshots). Raw (as-collected URL records) and Processed (unified feature table, official splits).
Data collection	Phishing URLs scraped with Python scripts from three public feeds: the NCSC “Tin Nhiem Mang” blacklist, the ChongLuaDao community blocklist (mirror snapshot, 2024-05-16) and OpenPhish. Legitimate URLs from the NCSC trusted-organisation registry and a Tranco top-list sample (global and .vn). Records normalised, de-duplicated, PII-redacted, featurised into the CompPhish-aligned 21-feature schema, scenario-labelled from URL brand tokens, tiered by source verification status, and split temporally by registrable-domain group. Page HTML/screenshots for a subset captured via urlscan.io sandbox.
Data source location	Institution: Campus in Ho Chi Minh City, University of Transport and Communications, Ho Chi Minh City, Vietnam. Primary public sources: https://tinnhiemmang.vn (NCSC, Vietnam); https://chongluadao.vn via its public mirror https://github.com/elliotwutingfeng/ChongLuaDao-Phishing-Blocklist ; https://openphish.com ; https://tranco-list.eu ; https://urlscan.io.
Data accessibility	Repository name: Mendeley Data
	Data identification number: DOI 10.17632/b97hxbxtpd.4
	Direct URL to data: https://data.mendeley.com/datasets/b97hxbxtpd/4
	Instructions for accessing these data: the open tier (URL tables, features, labels, splits and documentation) is publicly available under CC BY 4.0 with no registration. The gated companion (captured phishing/benign page HTML and screenshots, which depict live brand-cloning pages) is available from the corresponding author on request under a research-only data-use agreement. Collection and processing code: https://github.com/vuthainguyen1602/phishvn (MIT).
Related research article	None

## 1 Value of the Data

• An open, *time-stamped* URL phishing dataset localised to Vietnamese targets, pairing a real NCSC blacklist feed with both an *easy* curated-whitelist negative and a *hard*Tranco top-list negative: a benign design that resists the trivial “trusted-vs-malicious” shortcut and yields a more realistic operating point.
• Includes *impersonation-scenario labels* (bank, government, tax, e-commerce, telecom, delivery, social, gaming) inferred from URL brand tokens and reflecting Vietnam-specific impersonation, useful for targeted and explainable detection. The inference names a scenario for ≈9% of phishing records (unbranded, random-string domains fall into other), so these labels are high-precision slices rather than full coverage.
• URL features are *schema-compatible with CompPhish*, supporting cross-dataset generalisation and feature-transferability studies.
• Provides *label-confidence tiers* (gold/silver, plus a community-reported bronze expansion) and a *group-aware, time-ordered*split (grouped by registrable domain) that prevents campaign-level leakage, supporting robust evaluation. Temporal ordering covers the dated groups: all verified-core phishing, the certified-organisation benign stratum, and almost all of bronze (each community entry is grouped under a registrable domain that mostly carries a reconstructed date, so the group inherits it); the web-whitelist and top-list negatives attest no event date themselves and their date-free groups are placed by deterministic hash (the split table quantifies each stratum’s temporally-placed share), so the time separation does not extend to the bulk of the negative class; drift studies should read it accordingly.
• Ships with reproducible URL baselines reported with *multi-seed spreads and bootstrap confidence intervals*, open collection/processing code, and a datasheet.
• A *preliminary* paired HTML/screenshot subset seeds future multi-modal work; the pipeline is already structured to attach page content and screenshots as coverage grows.
• Beyond detector training, the tiers plus the released audit instruments (label-audit codebook and blinded sheets, token-filter audit sample, per-seed prediction files) make the corpus usable for *label-noise and weak-supervision research*; and the negative-stratum recipe (certified registry + local top-list + global hard negatives) ports to any country that maintains a trust registry, as a template for building further localised corpora.

## 2 Background

PhishVN was compiled because no public Vietnamese phishing-website corpus existed. The URL datasets that drive phishing-detection research (PhiUSIIL [2], GramBeddings [3], URL-Phish [13], CompPhish [1]) are overwhelmingly English-dominant [14], and the one prior Vietnamese phishing study [18] did not release its data. Vietnam-relevant phishing URLs circulate only as operational blocklists (the authoritative NCSC *Tin Nhiem Mang* registry [6] and the ChongLuaDao community feed [8]), which supply no benign class, no confidence tiers, no feature table and no leakage-controlled split. The URL records themselves are therefore *secondary* data: state and organisational observations that this work did not generate. What is original here is the layer built on top of them: the unified schema, the label-confidence tiers, the scenario inference, the reconstructed first-seen dates, the registrable-domain-grouped temporal split and the Vietnamese-targeting token filter, together with the 868 sandbox page captures, which are primary. Meanwhile, Vietnamese corpora exist for neighbouring abuse tasks (spam reviews [15], clickbait [19], spam email [17]), and open Vietnamese generative models such as PhoGPT [16] lower the cost of producing fluent Vietnamese lures, increasing the need for locally grounded evaluation data. PhishVN therefore combines, on the same records, Vietnamese impersonation scenarios, gold/silver/bronze label-confidence tiers, first-seen timestamps, a registrable-domain-grouped temporal split, and URL features kept schema-compatible with CompPhish for cross-dataset study. A small paired HTML/screenshot companion seeds future multi-modal extensions.

## 3 Data Description

The Mendeley Data repository (DOI 10.17632/b97hxbxtpd.4) contains the **open tier**, PhishVN_v3.1.0_open.zip, and the **benchmark evidence bundle**, PhishVN_v3.0.0_results_bundle.zip; a companion **gated tier**, PhishVN_v2.0.0_pages_GATED.zip (page captures, unchanged since v2.0.0), is distributed on request (see *Data accessibility*). Table 1 lists every folder and file; the subsections below describe each file’s contents in turn.

**Table 1:** Repository structure: every folder and file in the deposited archives.

Path	Size/count	Contents
*Open tier*: PhishVN_v3.1.0_open.zip		
README.md	n/a	Overview, composition counts, usage notes
LICENSE	n/a	CC BY 4.0 licence text
CITATION.cff	n/a	Machine-readable citation metadata
MANIFEST.txt	n/a	SHA-256 checksum and byte size of every file
data/dataset_url.csv	53,116 rows	Full record table (29 columns)
data/vn_compphish.csv	53,116 rows	CompPhish-aligned feature table (28 columns)
data/splits/url_train.csv	36,909 rows	Official training split
data/splits/url_val.csv	7266 rows	Official validation split
data/splits/url_test.csv	8941 rows	Official test split
data/abuse_type.csv	36,706 rows	Rule-derived abuse type per phishing URL
data/attacks/p3_paraphrase.csv	386 rows	LLM-paraphrase evaluation set (text channel)
data/attacks/p3_paraphrase_band.csv	386 rows	The same set with paraphrase-strength bands
docs/datasheet.md	n/a	Datasheet (*Datasheets for Datasets*[4])
docs/schema.md	n/a	Column-level data dictionary
docs/data_sources.md	n/a	Per-source provenance, licence and legal notes

*Benchmark evidence*: PhishVN_v3.0.0_results_bundle.zip, all under p1a_results_bundle/		
per_seed_metrics.csv	16 rows	Metrics per model × seed
predictions/	18 files	Per-row scores: model × seed, difficulty, ablation
models/	5 files	Fitted seed-0 estimators (joblib)
environment.json; README; MANIFEST	n/a	Pinned versions, protocol, SHA-256

*Gated tier*: PhishVN_v2.0.0_pages_GATED.zip (on request)		
HANDLE_WITH_CARE.txt	n/a	Handling and redistribution instructions
pages/phishing/html/	209 files	Rendered phishing pages, <id>.html
pages/phishing/shots/	209 files	Phishing page screenshots, <id>.png
pages/benign/html/	659 files	Rendered benign pages, <id>.html
pages/benign/shots/	659 files	Benign page screenshots, <id>.png

### 3.1 data/dataset_url.csv: the full record table

One row per URL, 53,116 records in total: an 18,997-record verified gold + silver core (2587 phishing from the national feed, 16,410 legitimate) plus a 34,119-record community/feed bronze expansion (33,823 ChongLuaDao domains, ∼50% carrying a reconstructed first-seen date, and 296 OpenPhish URLs). The 29 columns fall into four groups:

• *Identity and provenance*: id (deterministic uuid5, also the file name of the gated page captures), channel, label(phishing/benign), source (tinnhiemmang, tinnhiem_web, tinnhiem_org, tranco, tranco_vn, chongluadao, openphish), lang, label_source, status (the source’s original handling status), tier (label-confidence: gold/silver/bronze) and split(train/val/test).
• *Scenario*: scenario (impersonation sector inferred from URL brand tokens: bank, gov, tax, ecommerce, telecom, delivery, social, gaming, other) and impersonated_org (named organisation where the source provides one; usually empty).
• *Timestamps*: collected_at (the event date attested by the source, i.e. the detection or certification date; empty for the web-whitelist and top-list benign strata) and scraped_at(when the row was fetched; provenance only, never used for splitting).
• *URL and basic features*: url, url_norm (scheme-stripped, lowercased), domain, tld, url_len, num_dots, num_subdomains, has_ip, has_at, suspicious_tld. The rank column carries the Tranco rank for top-list rows (empty otherwise); the remaining columns (text, msg_len, gen_model, has_url, is_llm) are reserved for planned text-message/email channels and are empty or zero-filled in this release.

### 3.2 data/vn_compphish.csv: CompPhish-aligned features

The same 53,116 URLs re-featurised into the exact CompPhish [1] schema: url, url_len, dom, dom_len, is_ip, tld, tld_len, subdom_cnt, letter_cnt, digit_cnt, special_cnt, eq_cnt, qm_cnt, amp_cnt, dot_cnt, dash_cnt, under_cnt, letter_ratio, digit_ratio, spec_ratio, is_https, slash_cnt, entropy, path_len, query_len, plus label, split and tier. All length/count/entropy features are computed on the *scheme-stripped* URL; is_https is retained for schema compatibility but is a collection artefact and is excluded from modelling, leaving a 21-feature modelling schema.

### 3.3 data/splits/: official split files

url_train.csv, url_val.csv and url_test.csv partition dataset_url.csv (same 29 columns) according to the group-aware temporal rule described in *Experimental Design*: 36,909, 7266 and 8941 rows respectively (70/15/15, grouped by registrable domain; dated groups ordered oldest → train, newest → test). Because the official files blend that temporal rule (for dated groups) with a deterministic per-group hash (for undated ones), Table 2 breaks the counts down per tier, source and assignment rule, so any sub-benchmark (a tier, a source, or the strictly-temporal subset) can be reconstructed exactly.

**Table 2:** Official split composition per label-confidence tier and source. “Temporal” is the share of rows whose registrable-domain group carries a source-attested first-seen date and is therefore placed by the temporal rule (oldest → train, newest → test); the remaining rows are placed by a deterministic per-group hash (70/15/15). Any sub-benchmark can be reconstructed from these strata.

Tier	Source	Label	Train	Val	Test	Temporal
gold	tinnhiemmang	phishing	1619	0	69	100.0%
gold	tinnhiem_web	benign	3362	271	2246	53.9%
gold	tinnhiem_org	benign	1659	9	358	100.0%
silver	tinnhiemmang	phishing	457	24	418	100.0%
silver	tranco	benign	4170	935	888	0.3%
silver	tranco_vn	benign	1813	346	353	0.8%
bronze	chongluadao	phishing	23,621	5623	4579	100.0%
bronze	openphish	phishing	208	58	30	27.7%

**Total**			**36,909**	**7266**	**8941**	78.5%

### 3.4 data/abuse_type.csv and data/attacks/: companion assets

abuse_type.csv types the positive class beyond brand impersonation: a rule-derived abuse type per phishing URL (brand_impersonation 3364; gambling 337; investment 284; betting_stream 40; adult 15; unknown 32,666, mostly unbranded community-reported domains). It makes explicit that much of the community-reported positive class is abusive-but-not-impersonation, complementing the impersonation-only scenario taxonomy. data/attacks/ holds a fixed 386-variant LLM-paraphrase evaluation set for the planned text-message channel (with a paraphrase-strength-banded copy), released so robustness results against it are reproducible; it contains no personal data.

### 3.5 docs/: documentation

datasheet.md documents motivation, composition, collection, preprocessing, uses and maintenance following the *Datasheets for Datasets* framework [4], and registers the inter-annotator label audit (blinded 200-row stratified sample, Cohen’s κ), whose instruments are released with the code; the audit’s result is reported in *Experimental Design* and recorded in the deposited datasheet. schema.md is the column-level data dictionary for both CSV schemas. data_sources.md records, per source, the acquisition method, record counts, licence and legal basis, and mitigation notes.

### 3.6 Gated tier: captured pages

The gated archive pairs 868 records of dataset_url.csv (209 phishing, 659 benign) with the page as rendered at capture time: pages/{phishing,benign}/html/<id>.html (the rendered DOM) and pages/{phishing,benign}/shots/<id>.png (a full-page screenshot), where <id> is the record’s id. HANDLE_WITH_CARE.txt states the handling rules (research-only; open HTML in an isolated VM; no redistribution). This companion covers ≈1.6% of URLs and seeds future multi-modal work; the capture instruments are described in *Experimental Design*.

### 3.7 Composition summary

**Fig. 1:**
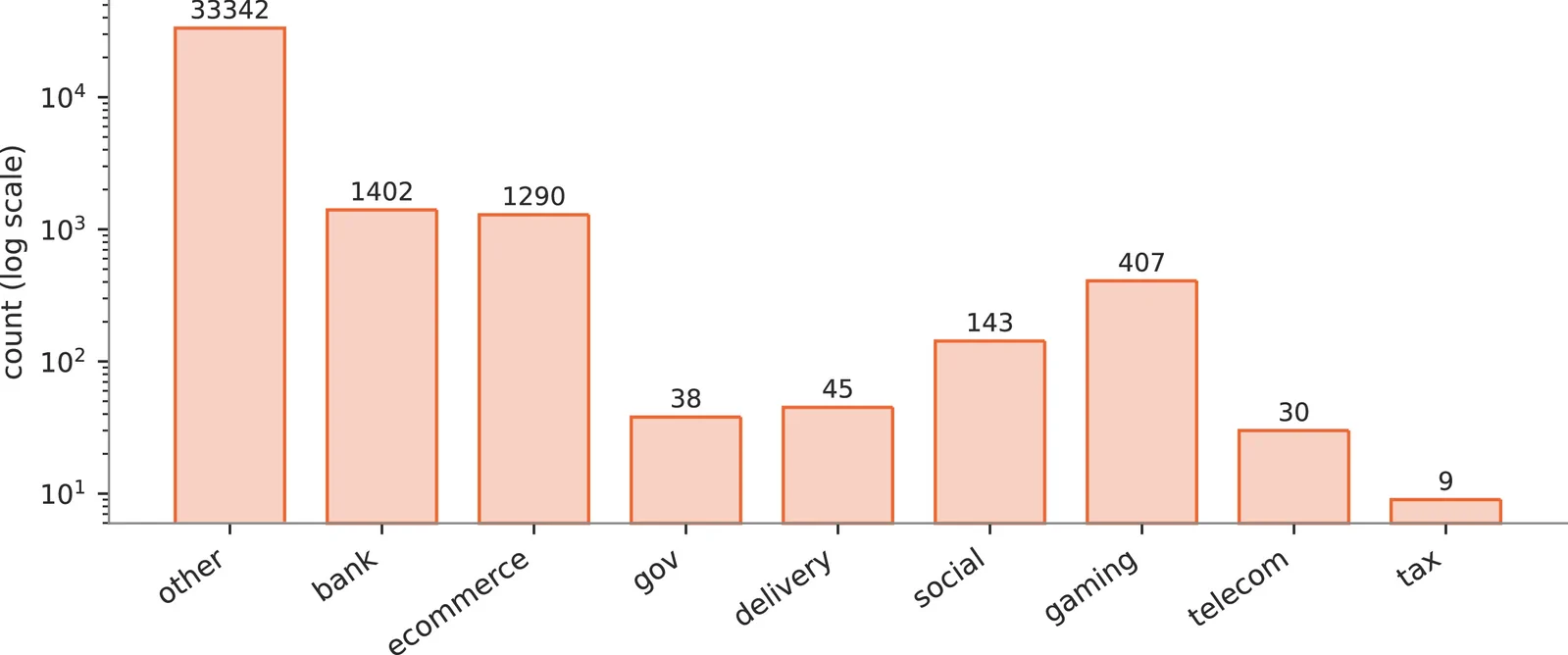
Phishing records by impersonation scenario, inferred from brand tokens in the URL (log scale; bar labels are real counts).

**Fig. 2:**
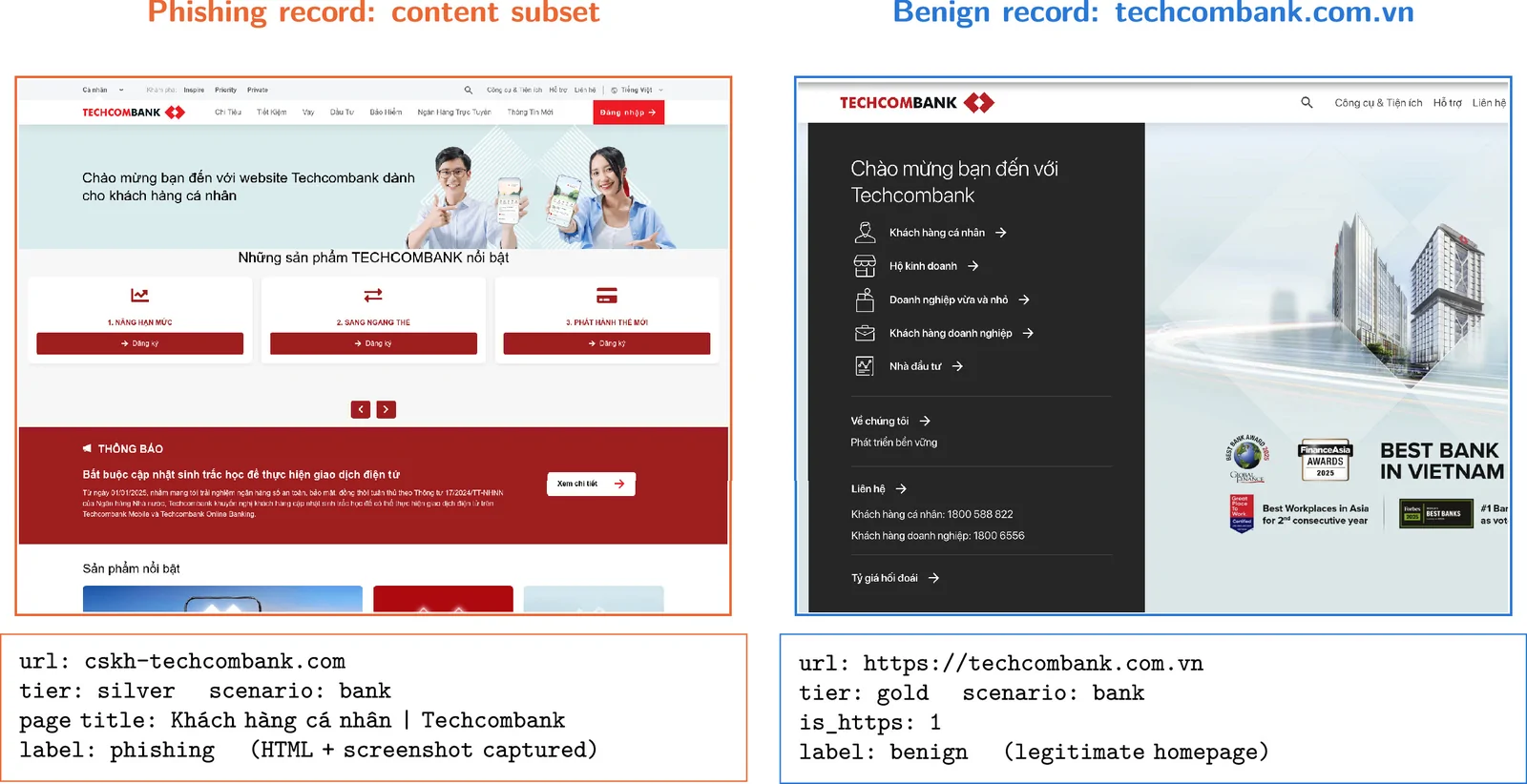
One released record pair, with its gated-tier screenshots cropped to page content. *Left:* a phishing record, cskh-techcombank.com (NCSC blacklist, bank scenario, silver tier). *Right:* the legitimate techcombank.com.vn.

Table 3 summarises records per label-confidence tier with phishing first-seen date ranges; Fig. 1 shows the phishing records per impersonation scenario, and Fig. 2 shows one released phishing/benign record pair with their captured screenshots. Unbranded, random-string domains carry no recognisable brand token and fall into other, the largest bin; among the branded lures, *bank* and *e-commerce* are the most frequent scenarios. The record pair in turn shows what the labels rest on: both sites render as well-formed Techcombank-branded pages, the phishing one being a visual clone of the legitimate site, so the record-level signals that separate them are the URL string, the provenance and the confidence tier.

**Table 3:** Dataset at a glance, per label-confidence tier; dates are source-attested phishing first-seen dates.

Tier	#Phishing	#Benign	Date range (phishing first-seen)
gold (handled/verified)	1688	7905	2020-02-08–2025-01-15
silver (under processing/feed)	899	8505	2021-05-27–2025-02-18
bronze (community/feed, ∼50% dated)	34,119	0	2021-05-07–2024-05-16

**Total**	**36,706**	**16,410**	53,116 records

Benign sources: tranco (5993), tinnhiem_web (5879), tranco_vn (2512), tinnhiem_org (2026). Splits: train 36,909, val 7266, test 8941. Gated content companion: 868 paired HTML + screenshot captures (209 phishing, 659 benign).

## 4 Experimental Design, Materials and Methods

### 4.1 Sources of the released URL table

The verified phishing core is scraped from the NCSC Tin Nhiem Mang blacklist [6] (scrape_vn_phishing.py); the bronze expansion adds the ChongLuaDao community denylist [8] (fetch_chongluadao.py, importing the public mirror snapshot of 2024-05-16, with per-domain first-seen dates reconstructed for ∼50% of domains by chongluadao_first_seen.py from Wayback-archived API ObjectIds and mirror git history) and the OpenPhish feed [9]. Benign URLs combine three source-tagged strata: the certified “trusted organisation” registry (scrape_trusted_orgs.py; curated, established .vn sites, *easy* negatives), a .vn-filtered Tranco [7] slice (popular sites Vietnamese users actually visit), and a global Tranco sample stratified across rank bands (fetch_tranco.py), whose long tail contributes lexically messier, *hard* negatives. Mixing easy and hard negatives prevents a classifier from exploiting a “trusted-vs-malicious” shortcut.

Every URL row in this release was acquired *retrospectively* from the sources above, and the table’s source column enumerates exactly them: seven values, the trusted-organisation registry contributing two (tinnhiem_web, tinnhiem_org); Table 2 accounts for every row against them. The crawl-at-detection pipeline described next is a *content-capture* instrument: its feed union (which also includes URLhaus [10] and the Phishing.Database project [11]) triggers page captures and accumulates candidate domains for future dataset versions, but contributes no URL row to the released table.

One consequence of the source formats deserves its own table. ChongLuaDao and Tranco ship bare domains by construction, and the NCSC feed also lists a bare domain in all but a fraction of its entries, so nearly every record in *both* classes has an empty path and query (99.5% of phishing, 99.9% of benign; Table 7); only OpenPhish (n=296) routinely carries full URLs. The path_len and query_len features therefore carry almost no signal of either class or source in this corpus, and the ablation reported with the reference benchmark (Table 8) verifies that dropping them changes nothing.

**Table 4:** Difficulty breakdown per stratum for the leaderboard’s best model (HistGB (grad. boosting), by mean PR-AUC) on the temporal test split. Intervals are Wilson score 95% CIs.

Stratum	Description	n	Value	95% CI
*Phishing detection recall by label-confidence tier (recall = TP/(TP + FN))*				
gold	trained core	69	0.681	[0.564, 0.779]
silver	trained core	418	0.770	[0.728, 0.808]
bronze	community expansion (unseen at train)	4609	0.885	[0.876, 0.894]

*Benign false-positive rate by negative stratum (FPR = FP/(FP + TN))*				
registry	certified trusted-org whitelist	2604	0.018	[0.014, 0.024]
.vn-popular	Tranco .vn top-list sample	353	0.025	[0.013, 0.048]
global hard-neg.	Tranco global top-list sample	888	0.502	[0.469, 0.535]

**Table 7:** Bare-domain share per source: records whose scheme-stripped URL has neither path nor query (path_len = query_len=0). Class totals: phishing 99.5%, benign 99.9%.

Class	Source	n	Bare-domain share
phishing	chongluadao	33,823	100.0%
phishing	tinnhiemmang	2587	99.6%
phishing	openphish	296	44.6%
benign	tranco	5993	100.0%
benign	tinnhiem_web	5879	100.0%
benign	tranco_vn	2512	100.0%
benign	tinnhiem_org	2026	98.9%

**Table 8:** Bare-domain ablation: the benchmark’s best model (HistGB (grad. boosting), seed 0, threshold 0.5) refitted without path_len and query_len under the identical protocol. Top: core-test metrics (gold + silver). Bottom: phishing recall per label-confidence tier on the full temporal test split, with Wilson 95% CIs.

Feature set	F1	PR-AUC	ROC-AUC	FPR@90%rec.
CompPhish (21 feat.)	0.543	0.626	0.923	0.180
- path_len, query_len (19 feat.)	0.543	0.626	0.923	0.180

Feature set	recall gold	recall silver	recall bronze	

CompPhish (21 feat.)	0.681 [0.564, 0.779]	0.770 [0.728, 0.808]	0.885 [0.876, 0.894]	
- path_len, query_len (19 feat.)	0.681 [0.564, 0.779]	0.770 [0.728, 0.808]	0.885 [0.876, 0.894]	

### 4.2 Content capture: retrospective crawl and crawl-at-detection

**Fig. 3:**
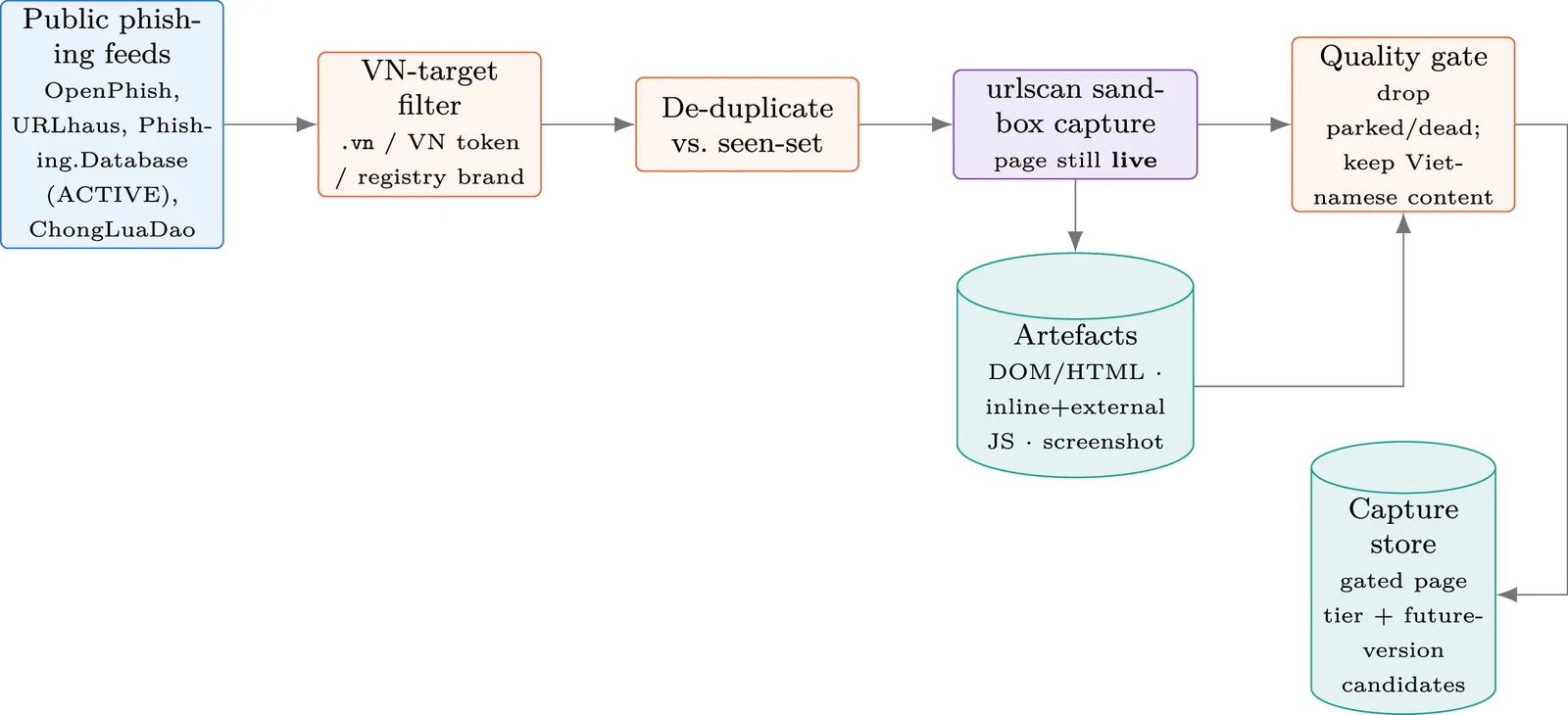
Crawl-at-detection collection: feeds are filtered to Vietnamese targets, de-duplicated, captured live in a sandbox, and quality-gated before entering the capture store that supplies the gated page tier and future dataset versions; the released URL table is assembled separately, from the retrospective sources.

Phishing infrastructure is short-lived: most phishing URLs are withdrawn within hours to a day of appearing, so a retrospective crawl of an aged blacklist inherits survivorship bias (in a content audit of live Vietnamese-named blacklisted domains, only about a fifth could be confidently identified as still-serving phishing). Two instruments therefore produced the gated page tier. An initial *retrospective* sandbox crawl captured blacklisted domains that were still live; the audit above is what quantified its bias. To escape that bias, the *crawl-at-detection* collector acquires page content *at detection time*: a domain is captured while still live, immediately after it first appears on a threat feed; it runs continuously and its captures accumulate for future dataset versions. Fig. 3 summarises the collector’s flow and Algorithm 1 gives the procedure.

**Algorithm 1:**
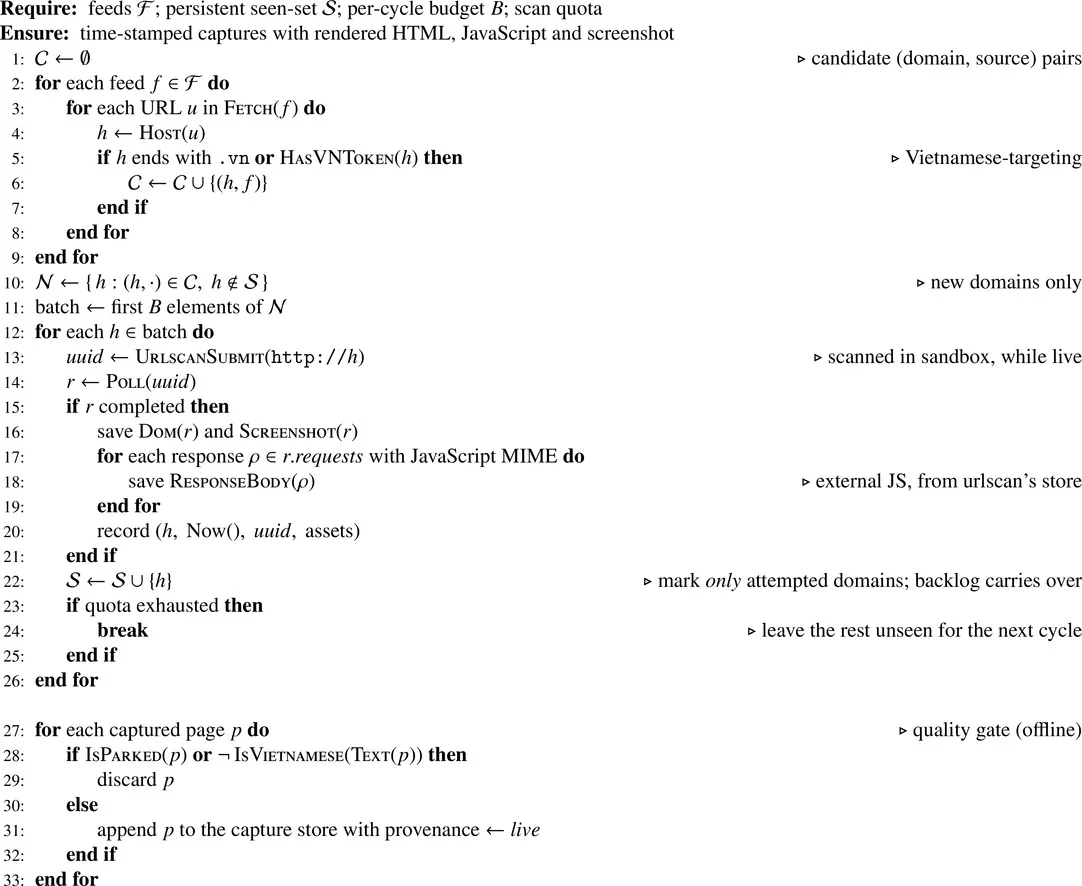
Crawl-at-Detection Vietnamese Phishing Collection (one polling cycle).

*Feeds and Vietnamese targeting* The capture pipeline unions four public feeds: OpenPhish [9], URLhaus [10], the freshness-filtered ACTIVE list of the Phishing.Database project [11], and the ChongLuaDao community denylist [8]. A domain is *Vietnamese-targeting* if it is under .vn *or* its name contains a Vietnamese unaccented token (a bank/wallet/authority/e-commerce/delivery term, e.g. vietcombank247, 247-napas, giaohang…). This name-level test is essential: under 4% of the Vietnamese phishing in our data uses a .vn TLD (3.7% of the verified NCSC set, 2.6% of the ChongLuaDao set); the rest hides on international TLDs (.com, .top, .xyz, …), which a TLD-only filter misses entirely.

The token list itself is two-tier. A hand-curated core covers the major brands, flagship government portals (e.g. the e-tax portal thuedientu.gdt.gov.vn, whose fused form thuedientu a whole-word thue pattern would miss), and the Vietnamese action vocabulary of phishing lures (dangnhap, xacminh, chuyentien, …). From July 2026 it is extended *automatically* from the NCSC trusted-organisation registry [6]: the certified real domain and unaccented name of each of its 7075 organisations yield 1798 additional brand tokens, so the filter’s brand coverage grows with the registry instead of with manual maintenance. Three guards keep global phishing out of the Vietnamese-targeting slice, and each dropped token is reported for audit. First, the registry also certifies Vietnamese *branches* of global brands (PayPal, JPMorgan, DHL, …) under their global domains; a token whose source organisations hold no .vn domain is dropped (440 tokens), since such a brand attracts *worldwide* rather than Vietnamese-targeting phishing. Second, a surviving token that still matches multiple popular non-.vn domains from the Tranco top list [7] is dropped as globally ambiguous (41 tokens; e.g. google matches 213 of the top 100k). Third, tokens shorter than six characters match only at word boundaries, preventing short fragments from firing inside unrelated names. On the unified feeds these guards matter: without them the extension would have flagged thousands of spurious “Vietnamese-targeting” domains (∼7900 on a 2026–07 feed snapshot; paypal-merchant.ru and similar); with them, on the audited snapshot of 2026-08-12, it grows the flagged set from 836 to 1115 domains (audit_token_filter.py). A seeded random sample of n=100 of the domains flagged only by the registry-generated tokens was manually audited under the same plausibility-is-not-evidence rule as the label audit: 9 were credibly Vietnamese-targeting (brand imitations or genuinely Vietnamese-language names), 84 were spurious matches and 7 could not be resolved from the name and public captures. On the resolved rows the extension’s precision is 0.10 (Wilson 95% CI [0.05, 0.17]).

Most of the audited false matches trace to two collisions the guards cannot see: a Japanese parent brand certified through its Vietnamese branch under a .vn domain (AEON, whose aeon.co.jp card-phishing campaigns dominate the sample), and a seven-character token that is a generic English suffix (ldesign, firing inside *floral design* and similar). This low name-level precision costs scan quota, not dataset purity: every flagged domain still faces the content-side quality gate of Algorithm 1, which keeps only Vietnamese-content captures, and no token-flagged domain enters the released URL table at all. The quota cost is worth stating rather than leaving as an aside. On the 2026-08-18 feed snapshot the registry tier is the sole basis for 277 admissions from the international feeds against 190 from the Vietnamese-reported one (a marginal precision of 0.41), and at the collector’s 960 submissions/day those 277 consume about 29% of one day’s budget, once. The concentration is extreme: a single certified token (aeon, whose Vietnamese subsidiary holds a .vn domain and whose Japanese parent attracts a card-phishing campaign) accounts for 110 of them against one Vietnamese-reported match, and 61 tokens contribute 135 admissions with no Vietnamese-reported match at all. Neither existing guard can see this: aeon matches no non-.vn domain in the Tranco top 100k, because the collision is with a phishing campaign rather than with a popular site. Records collected before this extension used the hand-curated core only.

*Safe capture* Each candidate domain is submitted to urlscan.io [5], which renders the page in *its* sandbox; no malicious page is ever loaded locally. The retrospective crawl used the same sandbox mechanics, so every released capture was rendered off-host. From the completed scan the pipeline retrieves the rendered DOM, the screenshot, and each JavaScript response body from urlscan’s stored responses (so even external kit scripts are captured without contacting the phishing host). Captures are private/unlisted and research-only.

*Backlog and budget* A persistent seen-set makes each cycle handle only unseen domains, and only the domains actually *attempted* in a cycle are marked seen, so a bounded per-cycle budget B drains a large initial backlog over successive cycles rather than discarding it, and a quota interruption simply resumes next cycle. On an edge device (an NVIDIA Jetson) the pipeline runs hourly under cron at B=40 (960 scans/day, within the API’s 1000/day quota), storing artefacts locally and serving a live monitoring dashboard on the LAN.

*Quality gate and provenance* Because even freshness-filtered feeds mix genuine phishing with parked pages and the occasional mislabelled business, an offline gate drops parked/dead captures and any page whose visible text is not Vietnamese. This is a language-and-liveness gate, not a label re-verification (a mislabelled Vietnamese-language page would survive it), which is one reason the store’s *URL candidates* are deferred to future versions and their label review, while its page captures supply the gated tier. Each surviving page carries a *live* provenance tag that distinguishes it from the retrospective crawl, so the two can be compared and the cleaner live set preferred as coverage grows.

### 4.3 Labelling

Labels are inherited from the sources: phishing URLs from blacklist entries, legitimate URLs from the certified registry and the Tranco top-list. The inherited labels are additionally curated by hand: a site-level label-conflict review excluded five ambiguous loan/shop .vn sites listed by one source and certified by another; twenty-one reviewed trusted-organisation misattributions were removed; and the re-scraped registry stratum was audited before release (2026-08-04). None of its 2026 benign rows appears on any phishing blocklist, and 88% are .gov.vn/.edu.vn/.org.vn domains, where registration itself requires the identity the benign label asserts. Scenario labels are assigned heuristically from brand tokens in the URL; because the feed ships impersonated_org empty, this token-based inference is the practical signal, and unbranded/random-string domains fall into other. To quantify label quality we registered an inter-annotator audit in which two annotators independently re-check a blinded 200-row stratified sample against a four-way verdict: credential *phishing*, other *scam* (gambling, betting streams, investment fraud, counterfeit shops, adult), *legitimate*, or *unsure*. The vocabulary is four-way because the dominant feed is an anti-fraud list rather than an anti-phishing one, so a binary verdict would force an abusive-but-not-phishing domain into the wrong cell and report the feed’s *scope* as if it were the feed’s *error*. The resulting agreement (Cohen’s κ) together with the consensus-versus-source label-noise estimate is reported below and recorded in the deposited docs/datasheet.md. The codebook fixes two decisions before annotation rather than after: what may be consulted (the corpus’s own captures, a web archive at the domain’s first-seen date, a public scan’s screenshot, or the trusted-organisation registry, but never a blocklist or reputation service, since an audit that consults the feed it audits measures only its own circularity), and that plausibility is not evidence, so a name that merely could belong to a real business is *unsure* rather than *legitimate*. Each row records which source decided it, and the resolvable share is reported beside κ: a noise estimate computed on the rows that evidence reached is not a claim about the ones it did not. The codebook, the sampling script and both annotation sheets are released with the code, so the instrument is auditable alongside its result. The audit is complete: both annotators covered all 200 rows. On the independent round, four-way agreement is 0.710 (Cohen’s κ=0.609); collapsed to the abuse-versus-legitimate-versus-unsure distinction the released binary label actually makes, agreement is 0.820 (κ=0.725); most disagreement is the phishing-versus-scam subtype (22 of 58 disagreed rows) or the evidence threshold against *unsure* (28), with only 8 rows contested across the abuse–legitimate line. Both annotators resolved 130 of 200 rows; documented arbitration of the 58 disagreements (23 resolved toward one annotator, 32 toward the other, 3 toward neither; one instrument amendment, a lookup-link correction, is recorded in the codebook) brings the final resolution to 149 of 200. Against the source labels, 12 of the 99 resolvable positive rows are *legitimate* by final judgment, a label-noise estimate of 12.1% (Wilson 95% CI 7.1–20.0%; 11 bronze rows, 1 silver), and 48 of the 50 resolvable benign rows are confirmed (noise 4.0%). Credential phishing is 42.5% of the defensible positives; the remainder is the anti-fraud scope the four-way vocabulary was designed to expose rather than misread as error.

### 4.4 Label-confidence tiers

Blacklist entries are all malicious; the source’s *handling status* is mapped to a tier rather than used to discard data: gold (status “handled/verified”), silver (“under processing”); weak-label “awaiting review” records are dropped by default. Curated benign sources are gold; the Tranco sample is silver. Community/feed records (ChongLuaDao, OpenPhish) are bronze. The primary benchmark trains on gold + silver; gold-only results are additionally reported as a robustness check on label confidence.

### 4.5 Processing pipeline

**Fig. 4:**
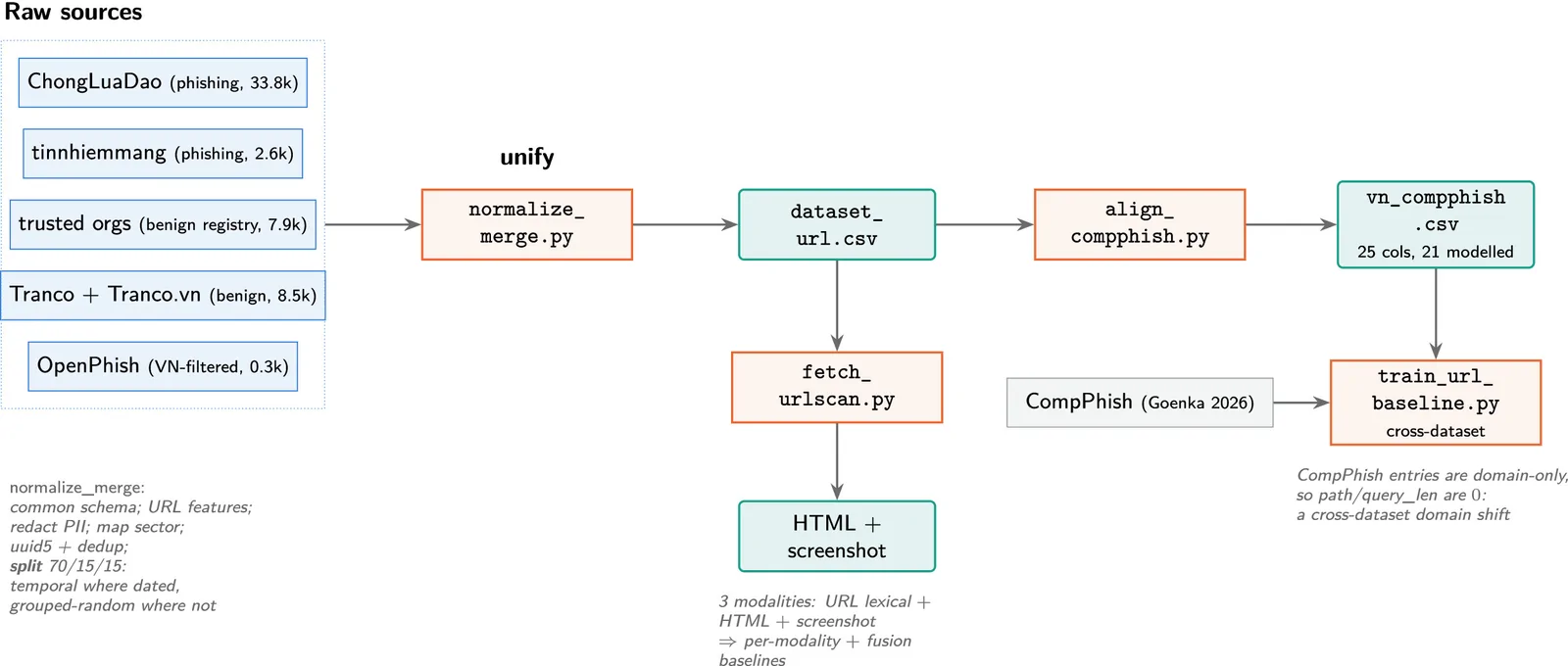
PhishVN data pipeline: heterogeneous raw sources are unified by normalize_merge, then re-featurised by align_compphish into the CompPhish schema.

Raw per-source CSVs pass through a two-stage, reproducible pipeline (Fig. 4). Stage 1 (normalize_merge.py) (i) maps each source to a common schema; (ii) extracts scheme-independent lexical URL features; (iii) redacts PII; (iv) infers the impersonation scenario from URL brand tokens; (v) assigns a deterministic uuid5 id and de-duplicates; and (vi) splits records *with grouping by registrable domain* (70/15/15), so that every subdomain of one campaign (e.g. hanoi.x.com.vn, hanoi3.x.com.vn) lands in the same split and cannot leak between train and test. The same grouping absorbs the few registrable domains that legitimately carry *both* classes: shared-hosting platforms whose apex is a popular benign site while user subdomains host phishing (github.io, pages.dev), and platform-page URLs blacklisted by the national feed under an otherwise benign domain. Each such group lands wholly inside one split, so a mixed-label domain can never straddle train and test. Each group takes as its timestamp the earliest collected_at among its members: the source-attested first-seen date, carried by the national-feed (gold + silver) core phishing, the certified organisations, and the ∼half of bronze ChongLuaDao domains whose first-seen date could be reconstructed. Groups that carry such a date are ordered temporally, train = oldest, test = newest. Groups with *no* event date on any member (in practice the top-list negatives, the web-whitelist rows whose group holds no dated record, and the undated remainder of bronze) are instead assigned to splits by a deterministic per-group hash (grouped random, 70/15/15), by design. The scrape date says nothing about the sample, is kept separately as scraped_at, and is never used for splitting. Table 2 reports the resulting split counts per tier and source together with the share of rows each assignment rule placed. Because the reconstructed ChongLuaDao dates participate in this ordering, they carry an accuracy estimate rather than an assumption. Validation of the reconstructed dates: 530 ChongLuaDao domains also appear on the NCSC blacklist, whose detection date is attested by the national feed rather than reconstructed. The difference (NCSC - reconstructed) has median 5 days (IQR 0 to 14), with 83% of pairs within 30 days and 93% within 90; since this difference also contains genuine inter-source detection lag, it upper-bounds the reconstruction error. An internal cross-check orders the reconstructed date against the domain’s first appearance in a downstream mirror’s git history (7477 domains; the mirror can only lag the database): 88% satisfy the ordering, and the 12% that do not miss it by a median of 5 days (99.9% within 30, maximum 65), reflecting day-level feed and commit jitter rather than misdating.

Stage 2 (align_compphish.py) re-featurises URLs into the CompPhish schema, so the URL table is directly interoperable with CompPhish; this is used for a single demonstrative transfer run, with the full cross-dataset study left to future work. One consequence of that re-featurisation is worth stating, because the pipeline figure shows it and nothing else here did: CompPhish records are domain-only, so path_len is 0 for 99.7% of rows and query_len for 99.9%. Two of the twenty-one features therefore carry almost no signal in the cross-dataset direction: a domain shift in the corpus, not a property of either detector.

### 4.6 Anonymisation

Phone numbers, emails, account numbers and names are redacted to tokens; any id↔PII mapping is kept private and encrypted, not released. Collection and release comply with Decree 13/2023/ND-CP [12] and Vietnam’s Personal Data Protection Law (effective 01/01/2026).

### 4.7 Reference benchmark

A single in-distribution reference benchmark is released on the CompPhish-aligned features: a four-model suite of Logistic Regression, Random Forest, gradient boosting and an MLP, with a Basic-schema Logistic Regression retained as a feature-schema ablation. Table 6 states every model’s architecture and hyperparameters, introspected from the released training code so the table cannot drift from what actually ran; per-seed metrics, per-row prediction files and the fitted seed-0 estimators are deposited with the dataset as PhishVN_v3.0.0_results_bundle.zip (generated by the released export_p1_results.py), so every interval below is verifiable without retraining. Metrics are F1, PR-AUC, ROC-AUC and FPR at 90% recall on the temporal test split. With TP/FP/TN/FN counted over the phishing (positive) class at the fixed threshold τ=0.5, and s(·) the model’s score,

(1) $$\begin{array}{cccc} F1 & =\frac{2\;TP}{2\;TP+FP+FN}, & \text{ROC-AUC} & =\mathrm{Pr}\;[s\left(x^{+}\right)>s\left(x^{-}\right)], \end{array}$$

(2) $$\begin{array}{cccc} \text{PR-AUC} & =\sum_{n}\left(R_{n}-R_{n-1}\right)\;P_{n}, & \text{FPR@90\%rec.} & =\mathrm{FPR}(\tau^{*}),\;\;\tau^{*}=\max\left\{\tau :R\left(\tau \right)\geq 0.90\right\}, \end{array}$$

where $P_{n},R_{n}$ are precision and recall at the n-th score threshold. Two details matter for reproducing these numbers: PR-AUC is average precision (the step-wise sum above), not a trapezoidal fit to the curve, and FPR@90%rec. is read at the *first* ROC point attaining 90% recall, so it is the lowest false-positive rate compatible with that recall. Per-stratum rates in Table 4 carry Wilson score intervals rather than Wald ones: for $\hat{p}=k/n$ successes and z=1.96,

(3) $$\frac{1}{1+z^{2}/n}\left(\hat{p}+\frac{z^{2}}{2n}\;\pm \;z\sqrt{\frac{\hat{p}\;\left(1-\hat{p}\right)}{n}+\frac{z^{2}}{4n^{2}}}\right).$$

Wald intervals misbehave exactly where this table lives: the smallest stratum has n=69 and one rate sits at 0.018, near the boundary. Models are compared by bootstrapping the F1 *difference* over the same resampled test rows, because two overlapping per-model intervals decide nothing either way; only the interval of the difference does.

**Table 6:** Reference-benchmark model configurations, introspected from the released training code (scikit-learn 1.4.2). Unlisted parameters take the library defaults; stochastic models are run over the listed seeds and the decision threshold is fixed at 0.5.

Model	Configuration	Seeds
Logistic Regression	penalty = l2, C = 1, solver = lbfgs, max_iter = 1000, class_weight = balanced	none (deterministic)
Random Forest	n_estimators = 300, criterion = gini, max_depth = None, min_samples_leaf = 1, max_features = sqrt, class_weight = balanced	0–4
HistGB (grad. boosting)	learning_rate = 0.1, max_iter = 100, max_leaf_nodes = 31, max_depth = None, l2_regularization = 0, early_stopping = auto, class_weight = balanced	0–4
MLP	z-score input scaling (StandardScaler), then hidden_layer_sizes = (128, 64), activation = relu, solver = adam, alpha = 0.0001, batch_size = auto, learning_rate_init = 0.001, max_iter = 400	0–4
Logistic Regression (Basic schema)	configuration as above, on the 9-feature Basic schema: url_len, num_dots, num_subdomains, has_ip, has_at, suspicious_tld, domain_len, num_hyphens, num_digits	none (deterministic)

Stochastic models are run over five seeds (mean ± std) and the best model (by PR-AUC) additionally carries a percentile bootstrap 95% confidence interval (B=2000) over the test set. Because these per-model intervals answer a different question from the one a leaderboard invites, we also compare the two leading models on *identical* test rows with a paired bootstrap of their F1 *difference*. No F1 is marked best. Scoring the two leading models (Logistic Regression, HistGB (grad. boosting); seed-0 fits) on identical test rows puts them −0.004 apart with a paired bootstrap 95% CI of [−0.025,0.016], which spans zero: the sign does not even survive the choice of seed, and this table therefore establishes no F1 ranking between them. Note that the per-model columns cannot answer this: overlapping marginal intervals neither imply nor deny a difference; only the interval of the *difference* does.

We stress this because overlapping per-model intervals are routinely (and wrongly) taken to settle a ranking in either direction. Table 5 reports the primary gold + silver benchmark; the bronze expansion stratum is excluded from this benchmark by construction (a community-reported scale/drift stratum) and is released for scale-hungry and drift studies. The two schemas trade places by metric rather than one dominating: the 9-feature Basic schema leads on the ranking metrics and threshold-dependent F1, while the full CompPhish schema delivers a markedly lower FPR at 90% recall, the operating point that matters most for a deployed filter. That the richer schema does not win outright is expected on this corpus: because both classes are almost entirely bare domains (Table 7), seven of the twenty-one CompPhish features (qm_cnt, eq_cnt, amp_cnt, under_cnt, slash_cnt, path_len, query_len) are constant on ≥99.3% of rows and carry almost no signal here, so the added width buys only the FPR improvement. An earlier build in which benign came only from the trusted whitelist and the stored scheme leaked the label produced a spurious F1 near 1.0; removing that artefact and adding 5993 hard Tranco negatives yields the operating points below.

**Table 5:** PhishVN reference in-distribution benchmark: a model suite on the canonical CompPhish temporal split (gold + silver, train n=13,080, test n=4332; ± is the standard deviation over seeds.

Model	F1	PR-AUC	ROC-AUC	FPR@90%rec.
*CompPhish schema (21 feat.)*				
Logistic Regression	0.539	0.589	0.917	0.180
Random Forest	0.463 ± 0.004	0.490 ± 0.002	0.878 ± 0.000	0.256 ± 0.002
HistGB (grad. boosting)	0.529 ± 0.009	0.619 ± 0.008	0.918 ± 0.004	0.210 ± 0.027
bootstrap 95% CI	[0.510, 0.575]	[0.583, 0.666]	[0.913, 0.932]	[0.164, 0.203]
MLP	0.416 ± 0.032	0.606 ± 0.019	0.908 ± 0.008	0.252 ± 0.024

*Basic schema (9 feat.): feature-schema ablation*				
Logistic Regression	0.633	0.665	0.926	0.244

Table 4 reads the best model’s performance (by mean PR-AUC) per source stratum: phishing detection recall per label-confidence tier above, benign false-positive rate per negative stratum below. That model is refitted on the verified gold + silver core alone (CompPhish features, fixed seed, threshold 0.5) and evaluated on the temporal test split, so the undated bronze community expansion it is scored on was never trained on: an out-of-core generalisation probe. Each rate carries a Wilson 95% interval on its own stratum’s n, necessary because the strata span two orders of magnitude in size, and the smallest of them (gold, n=69) carries an interval about twelve times wider than the largest, so the comparisons below are read from the intervals rather than from the bare rates. The global hard-negative (Tranco) stratum drives almost all false positives, while the curated .vn negatives are near-trivial. Community-sourced bronze phishing (never seen at training time) is recovered above both verified tiers with non-overlapping intervals, while gold and silver do not separate: at n=69 the gold interval spans 0.21.

A gold-only run (heavier class imbalance, test phishing rate 0.026; this slice also excludes the silver-tier Tranco hard negatives) is reported as a robustness check: the same model refitted on gold alone reaches ROC-AUC 0.98 with F1 0.54 under the imbalance.

These tables are strictly single-dataset; a single demonstrative cross-dataset run (train PhishVN → self-featurised CompPhish schema) illustrates schema interoperability.

The sources ship URLs at different granularities (ChongLuaDao and Tranco bare domains, the NCSC feed occasionally full URLs), so we verify that path_len and query_len measure the class rather than the source: Table 7 gives the bare-domain share per source (both classes sit at ≈99.5–100%), and Table 8 refits the best model without the two features under the identical protocol. Dropping path_len and query_len moves the bronze - gold recall gap from +0.204 (21 features) to +0.204 (19 features): the gap is essentially unchanged, so the bare-domain composition of the sources does not explain it. The two rows agree to every digit because the fitted model places no split on either feature (both are zero on over 99.6% of rows), so the ablation is confirmatory, and the released per-row predictions for the two fits are score-identical on the test split.

The tier contrast is therefore not an artefact of the sources’ URL formats. It remains partly confounded with time window and training exposure (gold test is only the 69 newest NCSC groups, Table 2), so we read it as consistent with community-reported lures being lexically more conspicuous than the national feed’s verified core, rather than as an isolated cause; the wide gold interval below is read in the same spirit.

## Limitations

The verified phishing core depends on the NCSC public feed, which has published no new detections after 2025-02-18 (verified 2026-07-14), so the gold/silver time axis currently ends there. The bronze stratum is community-reported and not independently verified; only ∼50% of its *domains* carry a (reconstructed) first-seen date, though because each blacklist entry is grouped under its registrable domain almost all bronze rows inherit a group date and are placed temporally, leaving only a small undated remainder to grouped random assignment. The released URL table is wholly retrospective: the crawl-at-detection collector contributes page captures and candidates for future versions, but no URL row of this release. Scenario labels are inferred heuristically from brand tokens in the URL, so unbranded or random-string domains fall into other (≈91% of phishing records) and token-less impersonation is not captured; impersonated_org is populated only where a source names the target, which is rare. The web-whitelist and top-list benign strata attest no event date (the certified-organisation stratum carries certification dates), which limits fully temporal evaluation to the dated groups. The gated HTML/screenshot companion covers only ≈1.6% of URLs (209 phishing, 659 benign pairs), so content-based modelling at scale is not yet supported. The is_https column is a collection artefact and must be excluded from modelling.

The filter’s recall is bounded below by its behaviour on a feed that is Vietnamese-targeting by provenance rather than by name. On the ChongLuaDao snapshot of 2026-08-18, 17,571 unique reported hosts yield 3022 admissions, so the filter discards 14,549 (83%) of a corpus every entry of which was reported by a Vietnamese victim. Manual inspection of the discarded remainder shows it is dominated by random-string gambling and betting infrastructure rather than credential phishing, so this bounds the filter and does not count missed phishing; brand imitations on neutral names (online-garena.com, maylanh.dien-may-xanh.net) do occur within it. The dataset’s phishing arm is therefore defined by a name-level rule, which a URL- or lexical-feature model evaluated on it inherits as a selection effect (audit_vn_filter_recall.py reproduces the measurement).

Finally, the dataset is localised to Vietnamese-targeting phishing; conclusions drawn from it may not transfer to other locales.

## Ethics Statement

The author has read and follows the ethical requirements for publication in *Data in Brief* and confirms that the current work does not involve human subjects or animal experiments, and that no content was crawled from social media platforms. Every record is drawn from a public blacklist, registry or top-list; where such a source lists a social-platform page URL as fraudulent, only that URL string enters the dataset. Page content is obtained from urlscan.io sandbox snapshots that only read the page (no form submission, no logins, no downloads), avoiding direct interaction with live malicious sites. Any personal data appearing in captured pages (phone numbers, emails, account numbers, names) is redacted to tokens; brand logos/marks are handled per fair-use for security research. Collection complies with Decree 13/2023/ND-CP and Vietnam’s Personal Data Protection Law (effective 01/01/2026). The captured phishing pages are distributed only in a gated, research-only tier under a data-use agreement.

## Declaration of Generative AI and AI-assisted Technologies in the Manuscript Preparation Process

During the preparation of this work, the author used Claude (Anthropic) in order to support manuscript restructuring to the journal template, drafting of the revision in response to peer review, and language editing. After using this tool, the author reviewed and edited the content as needed and takes full responsibility for the content of the published article.

## CRediT authorship contribution statement

**Thai Nguyen Vu:** Conceptualization, Methodology, Software, Investigation, Data curation, Validation, Visualization, Writing – original draft.
